# When Lubricant Retention Increases Ice Adhesion: Ionic Liquids in SLIPS

**DOI:** 10.3390/polym18172116

**Published:** 2026-08-31

**Authors:** Alexandre M. Emelyanenko, Timofei V. Golubitchenko, Vladimir G. Krasovsky, Kirill A. Emelyanenko, Ludmila B. Boinovich

**Affiliations:** 1 Frumkin Institute of Physical Chemistry and Electrochemistry, Russian Academy of Sciences, 119071 Moscow, Russia; ame@phyche.ac.ru (A.M.E.); volt.nemo@mail.ru (T.V.G.); emelyanenko.kirill@gmail.com (K.A.E.); 2 N.D. Zelinsky Institute of Organic Chemistry, Russian Academy of Sciences, 119991 Moscow, Russia

**Keywords:** ice adhesion, anti-icing, imidazolium ionic liquids, thermophysical properties, wetting

## Abstract

Slippery liquid-infused porous surfaces (SLIPS) can reduce ice adhesion, but their durability depends on coupled wetting, rheological, and phase-transition effects. Here, seven imidazolium ionic liquids (ILs) with varied alkyl and disiloxane substituents were evaluated as lubricants for laser-textured superhydrophobic aluminum. Surface tension, viscosity, thermal behavior, lubricant retention, wetting, and ice adhesion at −10 °C were correlated over 30 icing-deicing cycles. All freshly prepared coatings were water-wettable but exhibited weak droplet pinning, with sliding angles of 1.4–7.5°, despite apparent water contact angles below 90°. This combination reflects the lubricant-mediated interface, for which droplet mobility is not determined by the static contact angle alone. The behavior of SLIPS with different lubricants diverged under centrifugal loading: low-viscosity ILs were depleted, whereas ILs that solidified under the applied cooling protocol were retained more effectively within the texture. Counterintuitively, greater lubricant retention produced higher ice adhesion because solidified ILs stabilized ice bridges within the surface relief. The lowest adhesion after cycling was obtained for the coating infused with [C_9_C_3_Si_2_Oim][NTf_2_], which after lubricant depletion restored a superhydrophobic state with a water static contact angle of 171.6 ± 1.6°. These results identify lubricant phase state and interfacial redistribution as key design parameters for durable anti-icing SLIPS.

## 1. Introduction

Ionic liquids (ILs) are salts that exist in the liquid state, and many of them are liquid at room temperature. ILs exhibit widely tunable physicochemical properties, including very low vapor pressure for many compositions, broad liquid-state ranges, and strongly structure-dependent viscosity, conductivity, thermal stability, and interfacial behavior. These properties support a wide range of practical applications, including environmentally benign solvents and media for nanomaterial synthesis [1,2], electrolytes for ion batteries and energy-storage systems, high-temperature heat-transfer fluids [3,4,5,6,7], and antimicrobial agents [8]. Tribological applications are of particular interest, including the use of ILs as electrically conductive lubricants for modern electric vehicles [9] and as materials for protection against icing and biofouling [10].

Although the first report on the synthesis of ethanolammonium nitrate [11] appeared more than 130 years ago, interest in the synthesis of new ILs remains strong, and new compounds continue to be developed. Unlike conventional salts, ionic liquids contain bulky, asymmetric organic cations and diverse anions that hinder the formation of a strong crystal lattice, allowing the material to remain liquid at relatively low temperatures. The diversity of ionic liquids arises from the possibility of tailoring their physicochemical properties to a specific application by changing the cation or anion structure.

Another important advantage of many recently synthesized ILs is their low crystallization and glass-transition temperatures [12], which makes them promising lubricants for anti-icing slippery liquid-infused porous surfaces (SLIPS) [13,14,15,16,17]. SLIPS consist of a textured or porous solid substrate infused with a lubricant that preferentially wets and remains within the surface texture. The resulting liquid interface can reduce contact-line pinning and mechanical interlocking of ice with the underlying roughness, thereby facilitating water removal and lowering ice adhesion. However, for anti-icing applications, the long-term effectiveness of SLIPS is critically dependent on the ability of the lubricant to remain within and on the surface of the texture while maintaining sufficient interfacial mobility.

Lubricant can be depleted by shear, gravity, contact with water, and repeated icing/deicing, whereas excessive immobilization or solidification can suppress the lubricating function of the infused layer. Lubricant-layer stability is governed by several coupled factors, including lubricant molecular structure [18,19], surface forces acting in films of different thicknesses [20,21], and the morphology of the supporting texture. Consequently, both lubricant properties and surface architecture play key roles in determining the durability and icephobic performance of SLIPS [22,23].

Despite growing interest in IL-based SLIPS, only limited data are available on ice adhesion to surfaces infused with different ionic liquids, and the relationships between IL structure, physicochemical properties, phase behavior, lubricant retention, and coating durability remain insufficiently understood. Of particular fundamental and practical interest is the evolution of ice adhesion strength on IL-infused slippery coatings and the retention of ILs in the infused layer under high substrate velocities that model the operation of slippery coatings in aviation and wind-energy systems.

In this work, we investigated a series of monocationic ILs based on bis(trifluoromethylsulfonyl)imide, with alkyl and dimethylsiloxane-containing substituents in the imidazolium cation. Their density, viscosity, surface tension, and wetting properties were determined, together with the stability of the IL layer in a laser-textured coating and the evolution of ice adhesion during cyclic testing. Comparison of ILs with different substituent lengths, chemistries, and different positions of the imidazolium ring provides insight into how the molecular structure and phase state of the lubricant affect the durability and anti-icing performance of SLIPS.

## 2. Materials and Methods

### 2.1. Materials

The ILs investigated and their abbreviated designations are listed in Table 1. The synthesis and quality-control procedures are described in [24]. Since the synthesized ILs were aimed at being used as lubricants for surfaces when working in an open atmosphere at high water vapor humidity, we did not perform additional drying of the obtained substances. Moreover, to obtain well-reproducible results, ILs were exposed to a humid atmosphere for at least one month prior to being impregnated into a textured substrate in order to saturate them with water. The thermophysical properties of ILs 6-6 and 6-3S were characterized in our previous study [19] and are included here for comparison.

Two types of substrates were used in the wetting studies: smooth muscovite mica and laser-textured plates of D16 aluminum alloy. To change the surface state of the substrates from hydrophilic to hydrophobic or superhydrophobic, trimethoxy-{3-[(2,2,3,3,4,4,5,5,6,6,7,7,8,8,8-pentadecafluorooctyl)oxy]propyl}silane, synthesized in the laboratory of Prof. A. M. Muzafarov, was used [25].

### 2.2. Preparation of Hydrophobic, Superhydrophobic, and IL-Infused Slippery Coatings

Muscovite mica plates with a size of 20 × 30 mm^2^ were cleaved immediately before modification to obtain a freshly exposed surface. A cross-linked fluorooxysilane monolayer was deposited on the plates by chemical vapor deposition, as described in [19].

Superhydrophobic substrates of D16 aluminum alloy with a size of 30 × 20 × 2 mm^3^ were prepared by nanosecond laser surface treatment followed by chemical vapor deposition of the fluorooxysilane. The preparation procedure is described in detail in [26]. In brief, the metal plates were raster-scanned with an IR laser beam (wavelength, 1064 nm; pulse duration, 120 ns; pulse repetition rate, 40 kHz; laser output power, 100 W; scanning speed, 1000 mm/s; scanning density, 150 lines/mm; peak fluence, 140 J/cm^2^; laser beam diameter at the 1/e^2^ level, 30 µm). After laser processing, ultrasonic cleaning in distilled water, and drying at 130 °C, the surface was activated by oxygen plasma for 5 min. Chemical vapor deposition of the fluorooxysilane was performed at 95 °C for 40 min. Subsequent heat treatment of the hydrophobized samples at 150 °C promoted the formation of cross-linked siloxane bonds, resulting in a robust superhydrophobic coating.

The superhydrophobic coatings described above served as the porous substrates for SLIPS preparation. Because of the high viscosity of the ILs and their slow spreading over the superhydrophobic surface, the substrates were infused with a 1:1 *v*/*v* solution of the corresponding IL in isopropyl alcohol. The solution readily filled the surface texture and, after solvent evaporation, formed a continuous lubricant layer cloaking the topographic asperities.

For wetting measurements, the samples were first stored horizontally in a fume hood for 24 h to remove isopropyl alcohol from the lubricant layer by evaporation and were then kept vertically for an additional 72 h to allow excess IL to drain from the surface. Samples used for ice-adhesion measurements required more thorough removal of excess IL to prevent lubricant expelled during centrifugation from contaminating adjacent specimens. After isopropyl alcohol had evaporated for 24 h, these samples were placed in a vacuum chamber at 200 Pa for 10 min and then centrifuged under 50× *g* for 2 min at 23 ± 2 °C.

### 2.3. Measurement Methods

Smooth muscovite mica was used to assess the wettability of a surface modified with a dense, chemisorbed, cross-linked fluorosilane layer while excluding the effect of roughness on the measured contact angle. The wettability of IL-infused SLIPS by water was studied after removal of excess lubricant.

Static contact angles on all substrate types, the surface tension of the ILs, and the surface tension of water droplets placed on SLIPS were measured using a custom-built setup [27]. The setup determines the parameters of the Laplace curve that best fits the profile of pendant or sessile droplets from digitally processed images acquired with a video camera having a spatial resolution of 3000 × 2208 pixels, 256 grayscale levels, and a pixel size of 5.2 × 5.2 µm^2^. The surface tension of pendant droplets was determined with an accuracy of 0.02–0.03 mN/m, and that of sessile droplets with an accuracy of 0.2–0.3 mN/m. The contact angle accuracy was 0.02–0.05°. Sliding or roll-off angles were measured with an angular resolution of 3′ using the setup described in [28]. Temperature-dependent surface tension was measured in a Binder MK53 climatic chamber (Binder, Tuttlingen, Germany), in which the temperature deviation did not exceed 0.3 °C.

Because quantitative determination of droplet surface tension from the Laplace-curve parameters requires the knowledge of liquid density, and the data on lubricant viscosity is necessary for the analysis of the resistance of SLIPS to repeated ice formation and detachment, the temperature dependences of IL density and viscosity were measured using an SVM 3001 Stabinger viscometer-densimeter (Anton Paar, Graz, Austria).

The shear strength of the ice/coating adhesive contact was measured using a laboratory-built centrifugal apparatus [29,30]. The apparatus allows the adhesion strength of 24 samples to be measured in a single experiment under identical conditions of ice formation, aging at the target subzero temperature after crystallization, and ice detachment. The apparatus was installed in a Binder MK53 climatic chamber (Binder, Tuttlingen, Germany), enabling ice formation and detachment at a preset temperature.

The surface temperature of the disk with the samples during cooling and temperature equilibration was monitored using the temperature logger iButton DS1922L F5 (Maxim Integrated, San Jose, CA, USA) with an accuracy of 0.5 °C. The experimental procedure was as follows. Samples bearing slippery coatings infused with different ILs were fixed around the perimeter of a rotating disk at the same distance from its center. Two polypropylene cylinders were placed on each sample, and 100 µL of water were poured into each cylinder. As shown below, all coatings had static contact angles below 90°. To prevent water leakage during filling, the samples were precooled to −25 °C. After filling, the chamber temperature was again lowered to −25 °C and maintained until the water crystallized, after which the temperature was set to −10 °C. The ice aging time at the target temperature was measured from the crystallization of the last sample. Because the introduced liquid volume was 0.1 mL (that is, the crystallization occurred in bulk water), the crystallization times in cylinders placed on coatings infused with different ILs differed by no more than 2 min. The shear ice adhesion strength τ for each coating was calculated from the centrifuge rotation frequency at which each ice-filled cylinder detached, using the relation τ = *m*(2πν)^2^*R*/*S*, where *m* is the mass of the cylinder with ice, ν is the rotation frequency at detachment, *R* is the distance between the cylinder center and the axis of rotation, and *S* is the contact area between the ice and the sample. Because both adhesive and cohesive failure were possible, the sample surfaces were inspected for ice residues, and cohesive failures were excluded from the statistical analysis of adhesion strength.

The centrifugal experiment is particularly relevant to modeling the wall shear stresses for wind turbines and high-speed aircraft propellers. Aerodynamic loading acts primarily through the gas–liquid interface, whereas centrifugal loading acts as a body force throughout the lubricant volume. Nevertheless, both mechanisms drive lubricant redistribution and depletion along the textured surface. The present rotational test therefore should not be interpreted as an exact aerodynamic simulation, but rather as an accelerated mechanical stability test in which lubricant layers of micrometer-scale thickness are subjected to shear-driving stresses comparable to or exceeding those expected from aerodynamic skin friction in the intended application environments.

The same centrifugal apparatus was used in an additional experiment aimed at independently assessing the retention/removal of the IL layers under the centrifugal loading resembling that applied during cyclic ice formation/detachment. In this experiment, described in Section 4.1 as “a centrifugal loading”, the separate set of SLIPS samples infused with ILs under study was prepared and subjected to the same experimental procedure as in ice adhesion measurements, but without the formation of ice. Briefly, 30 cycles of SLIPS sample centrifugal loading were performed. In each cycle, the samples fixed at the rotation disk were precooled to −25 °C followed by a temperature rise to −10 °C. After 1 h equilibration at −10 °C, the disk was rotated with constant angular acceleration to 5000 rpm and stopped. The climatic chamber was warmed to room temperature for 30–40 min, and then the next cycle of centrifugal loading started.

## 3. Results and Discussion

### 3.1. Surface Properties of the Investigated ILs

As discussed in the literature [31], a rough substrate must be readily wetted by the lubricant to produce SLIPS that are uniform and stable during storage. However, wetting of a smooth surface does not exclude a metastable nonwetting state on a rough surface of the same chemical composition. Such a state may occur for highly viscous lubricants with low vapor pressure. For example, pseudo-nonwetting of a superhydrophobic substrate was observed for a dicationic bis(trifluoromethanesulfonyl)imide ionic liquid containing a hydrocarbon linker between the imidazolium rings [19]. In this case, infusion of the superhydrophobic substrate with an IL solution in a low-viscosity solvent promotes rapid spreading and yields a uniform lubricant layer after solvent evaporation [19].

For thermodynamically favorable wetting of a porous substrate, the Young contact angle on a smooth surface of the same chemical composition must be below 90° [32]. Therefore, static contact angles of droplets of different ILs were measured on smooth hydrophobic mica coated with fluorosilane. Table 2 presents the IL’s surface tensions measured by the pendant drop method at *T* = 25 °C and the contact angles measured by the sessile drop method at *T* = 23 ± 2 °C. The table also includes the properties of water droplets on SLIPS infused with the corresponding ILs.

The data in Table 2 support the following conclusions.

The surface tensions of all investigated ILs range from 25.18 to 30.68 mN/m. At *T* = 25 °C, a siloxane substituent decreases the surface tension by approximately 3.5–4.0 mN/m relative to corresponding dialkyl-tail liquids (compare 9-3S vs. 9-6 and 6-3S vs. 6-6 ILs). For 9-1 and 12-1 ILs containing one long-chain and one methyl substituents, the surface tension is approximately 1.5 mN/m higher than for liquids with two long chains in the cations. This difference may arise from less effective hydrophobic shielding of the ionic moiety.

The minimum value, 25.18 mN/m, was obtained for a monocationic IL 9-3S with the imidazolium ring in a central position and one alkyl and one dimethylsiloxane-containing tail. The maximum value, 30.68 mN/m, was observed for a monocationic IL 12-1 with a terminal imidazolium ring and the longest alkyl tail.

All investigated ILs wet the hydrophobized mica, with static contact angles ranging from 70.5° to 81.8°. Because these values are below 90°, wetting of the laser-textured superhydrophobic substrates and the formation of a continuous IL layer can be expected. The contact angle changes only moderately with IL chemical structure. Within the investigated series, larger contact angles generally correspond to ILs with lower surface tension, although the trend is not strictly monotonic.

Direct comparison of the measured values (Table 2) with literature data is difficult because no corresponding data are available for mica hydrophobized with the same fluorinated silane. For PTFE, the contact angle of IL 9-1 was reported as 64° in [33] and 88.9° in [34]. Because the chemical composition of the surface layer on the hydrophobized mica is similar but not identical to that of PTFE, these values can only serve as a reference. The contact angle measured here, 76.8 ± 0.9°, lies between the published values.

### 3.2. Temperature Dependence of the Thermophysical Properties of the ILs

The preceding discussion concerned ILs’ surface tension at room temperature. For anti-icing applications, however, surface tension, density, and viscosity over a broad temperature range, including subzero temperatures, are important. The temperature dependences of these properties were therefore investigated. To enable a more detailed analysis of the effect of IL structure on ice adhesion to IL-based SLIPS, ILs 6-6 and 6-3S were included in the analysis and listed in Table 1. Their thermophysical properties were determined previously [19], whereas the anti-icing properties of SLIPS based on these ILs had not been studied.

**Figure 1:**
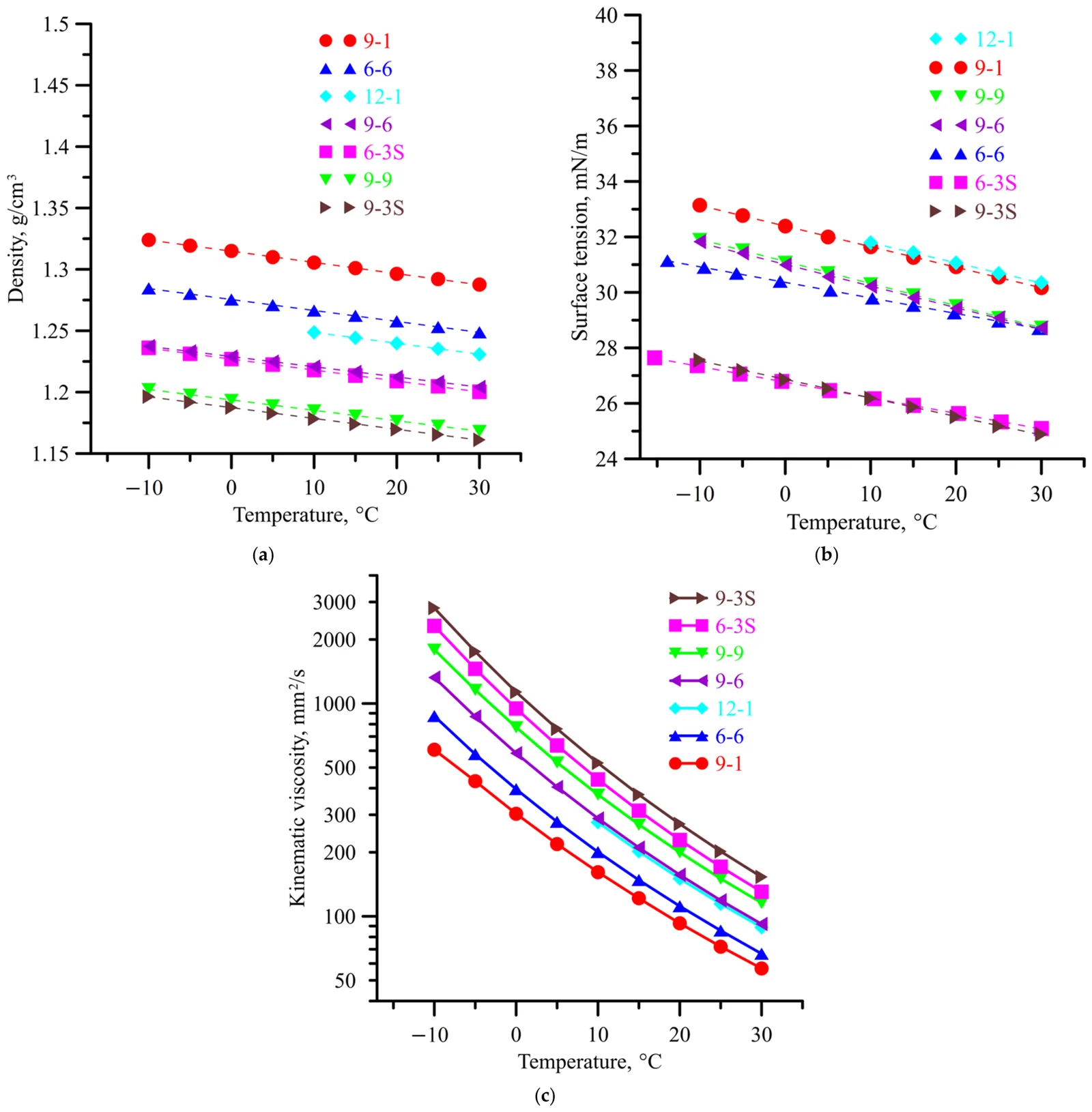
Temperature dependences of density (**a**), surface tension (**b**), and kinematic viscosity (**c**) for the investigated IL series. Dashed lines in (**a**,**b**) are linear fits; solid lines in (**c**) connect the experimental points to guide the eye.

For all investigated ILs, density varies linearly with temperature over the studied range (Figure 1a). Increasing the hydrocarbon-tail length for cations with a terminal imidazolium ring is also accompanied by a shift in the glass-transition temperature toward higher temperatures [34]. In addition, replacement of dimethylsiloxane groups with alkyl groups or extension of the hydrocarbon tail while retaining the overall cation architecture increases the IL density throughout the studied temperature range.

The temperature dependences of surface tension for all ILs except 12-1 are shown in Figure 1b over the range from −10 to +30 °C. For IL 12-1, the surface tension was measured from +10 to +30 °C. Upon further cooling, this liquid does not readily remain supercooled and crystallizes easily. The surface tension dependences in Figure 1b are well described by linear functions of temperature, with coefficients of determination close to 1. The temperature coefficients of surface tension are small for all ILs: even at *T* = −10 °C, the surface tension remains below 33.5 mN/m.

The kinematic viscosity of investigated ILs decreases sharply upon heating, as is typical of ionic liquids [19]. Between −10 and +30 °C, the viscosity of each sample decreases by approximately one order of magnitude. This behavior indicates thermally activated flow, which is apparently associated with temperature-dependent rearrangement of the ionic and hydrophobic fragments. The curves do not intersect, and therefore the relative viscosity ranking is retained throughout the investigated temperature range.

The highest viscosities are observed for the two ILs containing a disiloxane substituent. Comparison of ILs 6-3S and 9-3S shows that increasing the length of the alkyl substituent in the siloxane-containing cation further increases viscosity. This observation is consistent with the view that a longer hydrophobic fragment increases van der Waals interactions and hinders collective rearrangement of the molecular environment under shear.

A similar trend is observed among the ILs without siloxane structural fragments. Dialkyl cations containing more methylene groups have higher viscosities than structures with one long alkyl tail and one methyl substituent. Thus, IL 12-1 is substantially more viscous than IL 6-6. Viscosity is therefore determined not only by total chain length but also by cation architecture: extended N-alkyl substituent may strengthen hydrophobic association and slow structural relaxation.

IL 9-1 has the lowest viscosity over the entire temperature range. Differences among the samples are most pronounced at low temperatures and decrease upon heating. This behavior is consistent with an increasing contribution of collective structural constraints upon cooling, when the energy barrier to molecular displacement increases for larger and more strongly associated cations.

Thus, the flow behavior of the investigated ILs is governed by at least two structural factors. The first is the increased volume and length of the hydrocarbon tails, which strengthens interchain interactions and increases viscosity. The second is the presence of a disiloxane fragment, which increases the effective hydrodynamic volume and slows bulk structural relaxation.

### 3.3. Wettability of IL-Based SLIPS by Water

**Figure 2:**
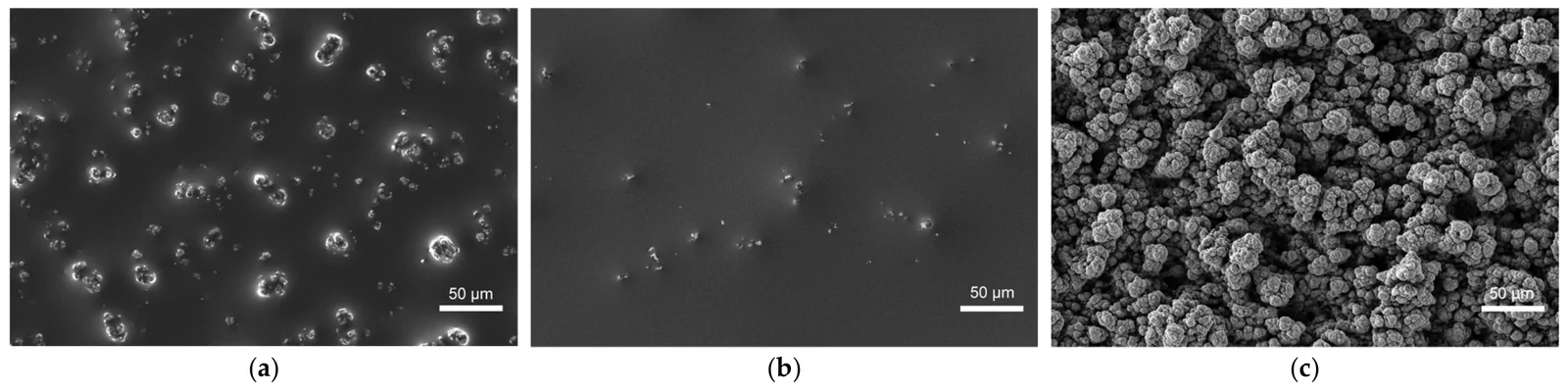
SEM images of the surface of SLIPSs infused with ILs 9-1 (**a**) and 9-3S (**b**) after drainage of excess IL by storage in vertical position and centrifugation under 50 g acceleration. Panel (**c**) shows the SEM image of non-infused superhydrophobic substrate for the comparison.

Infusion of the laser-textured superhydrophobic aluminum substrate with an excess of IL solution in isopropyl alcohol followed by alcohol evaporation produces a thick, smooth, wetting IL layer that covers the surface asperities. During the vertical storage, part of the liquid drains under gravity. The thickness of the lubricant layer remaining on the surface is governed by surface forces and is nonuniform when the sample is held vertically [20,35]. The amount of IL retained in the surface depressions is governed by capillary forces and therefore by surface morphology [36]. After 3 days of vertical storage, the SLIPS surface became moderately rough. Roughness was also observed on samples from which excess lubricant had been removed by centrifugation. SEM images of coatings infused with ILs 9-1 and 9-3S are shown in Figure 2.

Because the initial substrate texture was identical, the larger amount of IL 9-3S retained after centrifugation is consistent with its higher viscosity (Figure 1c).

The water contact angles of all freshly prepared SLIPS are below 90° (Table 2), indicating the hydrophilic character of the IL-covered surfaces. Despite the observed roughness, the sliding angles remain low. Water droplets can therefore be removed even at a small surface inclination. The absence of pronounced droplet pinning during sliding indicates that the asperity tops are covered by an IL film that provides a slippery response.

After contact with SLIPS, the surface tension of the test water droplets decreases for every investigated IL. This observation is consistent with the transfer of part of the IL to the water–air interface and the formation of a cloaking film, reflecting strong intermolecular interactions between water and the imidazolium moiety of the IL. Such transfer may accelerate lubricant depletion during repeated contact of the coating with water droplets.

The position of the imidazolium ring affects the IL–water interaction. A terminal polar imidazolium moiety produces a larger decrease in water surface tension. When the ring occupies a central position, long alkyl substituents may sterically hinder the approach of water molecules to the ionic moiety. Weaker interaction with water corresponds to larger water contact angles on SLIPS. For example, the static contact angle is 65.2° for the coating infused with IL 12-1 and 82.2° for the coating infused with IL 9-9. Siloxane groups in the tail also increase the water contact angle, consistent with their hydrophobicity.

## 4. Ice Adhesion to IL-Infused Slippery Porous Coatings

### 4.1. Factors Controlling Lubricant Retention and Removal

A conventional SLIPS architecture consists of a textured or porous solid substrate infused with a lubricant. The lubricant fills the pores and texture and forms a film over the asperity tops. For stable coating operation, the infused liquid must wet the substrate preferentially over external phases such as water, frost, or ice. The lubricant should also have low miscibility with water and maintain a mobile interface. Because the aluminum texture was fluorooxysilane-modified before infusion, lubricant retention in the present system is governed by IL interaction with the modified surface rather than by direct IL–aluminum adhesion.

According to extensive literature data, the architecture of anti-icing SLIPS reduces ice adhesion through several mechanisms [22,37]. First, mechanical interlocking with the rough substrate is substantially reduced because the surface depressions are at least partly filled with lubricant, thereby decreasing the real ice/coating contact area. Second, contact line pinning is reduced because the three-phase line is a liquid–liquid–air or ice–liquid–air contact line rather than a texture–ice junction. Third, when the lubricant layer is sufficiently thick, the shear stress required to remove ice is reduced by flow or slip within the lubricant layer. Finally, after local damage or displacement of the infused liquid, capillary forces redistribute lubricant along the surface, including toward the damaged region, partially restoring the slippery response to liquid and solid atmospheric precipitation.

When SLIPS are used to protect wind turbine blades or aircraft components from icing, aerodynamic loading limits the lubricant layer thickness, even for highly viscous lubricants and micro- or nanotextured surfaces. Wind-induced air friction shear stresses on wind turbine blades or aircraft propellers are of the order of tens to hundreds Pa (See the estimations in the Supplementary Information) and can therefore overcome the capillary pressure in pores hundreds of micrometers in size and remove lubricant from these pores. Removal of IL from large pores was also observed when samples were rotated on the disk of the ice adhesion apparatus (see Section 2). After these loads, the coating surface became markedly rougher (Figure 2).

**Figure 3:**
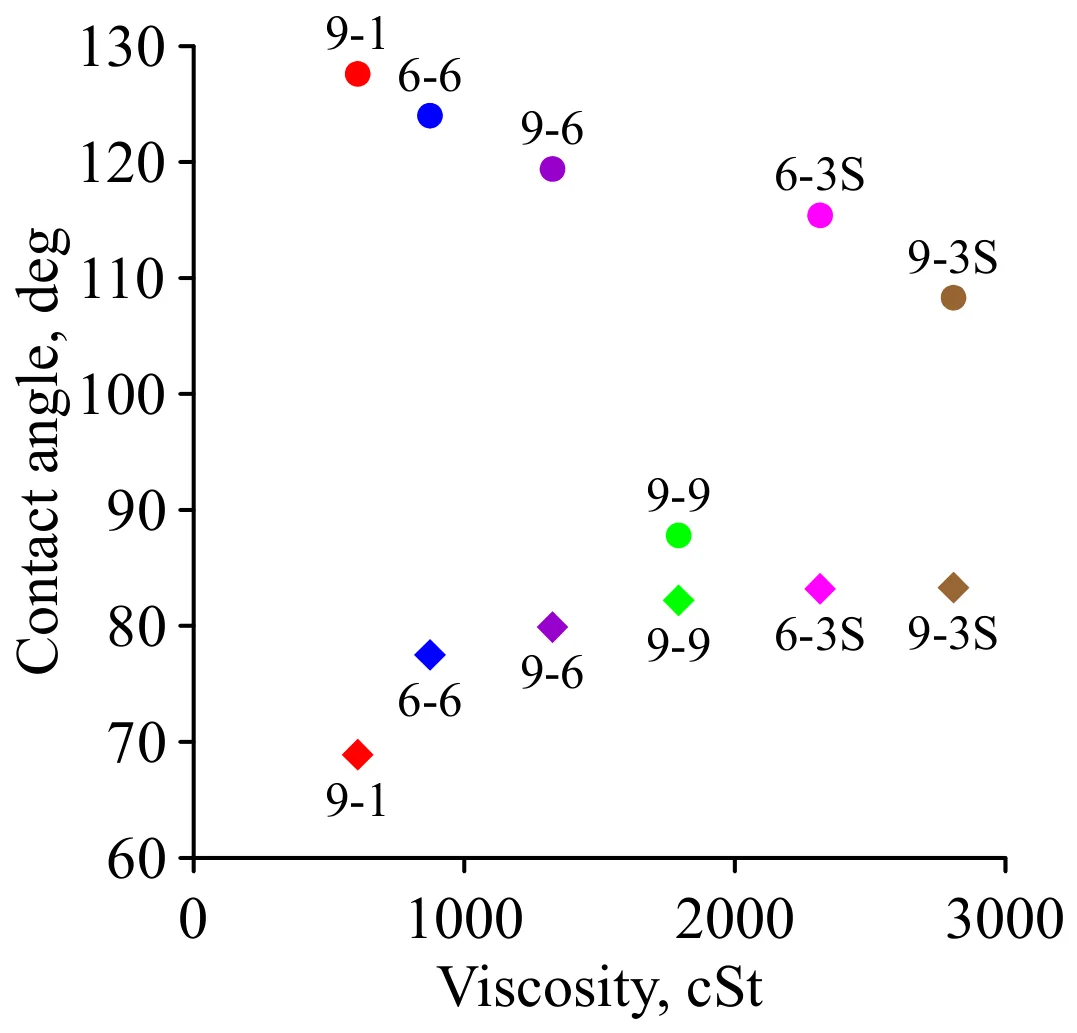
Correlation between the static contact angles of SLIPS before (diamonds) and after (circles) centrifugal loading and the viscosity of the corresponding IL.

To evaluate the stability of the IL layer, static contact angles were measured on samples infused with different ILs after prolonged centrifugal loading (Figure 3). The samples were positioned approximately 12 cm apart from the rotation axis and subjected to 30 acceleration cycles up to 5000 rpm at −10 °C, matching the configuration used for the ice adhesion measurements. During rotation, part of the IL was removed by centrifugal force.

In Figure 3, the contact angles measured before and after 30 cycles for each IL-infused SLIPS are plotted versus the viscosity of the corresponding IL at *T* = −10 °C. After loading, the contact angles differed substantially from their initial values, and the change was greater for the less viscous ILs.

A key feature of the contact angles measured after repeated centrifugal loading is that, after 30 cycles, the values exceed 90°. Thus, centrifugal loading transforms the initially hydrophilic SLIPS into a hydrophobic surface. Because the ILs were introduced into an initially superhydrophobic texture, the increase in contact angle indicates rupture of the IL film over the asperity tops and partial exposure of the superhydrophobic substrate. The persistently reduced surface tension of the test water droplets shows that some IL remains in the surface grooves.

The stability of the wetting film over the texture tops is one of the factors governing SLIPS behavior [20,21]. This stability depends on surface forces within the thin film, which are described by the disjoining pressure isotherm. After repeated centrifugal loading, droplets no longer slid from coatings infused with ILs 9-1, 6-6, 9-6, 6-3S, and 9-3S. This observation is consistent with the formation of a mosaic surface containing residual hydrophilic IL domains and exposed superhydrophobic regions. According to the Cassie law, the contact angle on such a mosaic surface is determined by the area fractions of the hydrophilic and superhydrophobic regions. Figure 3 shows that the largest contact angle after loading was obtained for SLIPS infused with IL 9-1, the least viscous liquid. This result indicates the largest exposed fraction of the superhydrophobic substrate after centrifugal loading.

Coatings infused with ILs 12-1 and 9-9 were exceptions to these trends. The viscosity of IL 12-1 at *T* = −10 °C was not measured because the liquid solidified. In the ice detachment and adhesion strength experiments, SLIPS were cooled in two stages, first to −25 °C and then warmed to −10 °C. Because both liquids have melting temperatures above 0 °C, their films transformed from a metastable supercooled state to a crystalline/solidified state. Solidification reduced IL removal by centrifugal force. Consequently, after loading, the contact angles of coatings infused with ILs 12-1 and 9-9 remained below 90° and differed only slightly from the initial values. At the same time, their sliding angles increased relative to the initial values (see Table 2), reaching 23.5 ± 3.4° for IL 12-1 and 56.8 ± 8.2° for IL 9-9.

### 4.2. Cyclic Ice Detachment Tests

Freshly prepared SLIPS infused with fluorinated lubricants or silicone oils have previously shown low shear ice adhesion strength [31,38]. However, repeated ice formation and detachment cycles caused lubricant loss, degradation of slippery properties, and increased adhesion [30,38]. Analysis of the effect of lubricant properties on ice and water adhesion to slippery surfaces showed that low-viscosity lubricants provide high mobility and initially low adhesion of aqueous precipitation, whereas more viscous oils are retained more effectively in the texture but restore lubricant-depleted regions more slowly [31,39,40].

The use of ILs containing polar groups has both advantages and limitations relative to silicone or fluorinated lubricants. Infusion with such ILs makes the SLIPS surface hydrophilic, as also observed in this work. Hydrophilicity increases the contact area of water droplets with the surface and may promote droplet retention and subsequent ice accumulation. At the same time, strong interactions between polar ionic fragments and water may produce bound water. As noted in several studies [41,42], a nonfreezing water interlayer may then form at moderately subzero temperatures and act as an additional lubricant layer.

Ionic liquids also have extremely low vapor pressures compared with many molecular oils, reducing evaporative losses. In addition, immobilization of ILs in ionogels, organogels, or polymer networks [16,43,44,45] can reduce macroscopic IL leakage while retaining interfacial mobility. Finally, changing the cation substituents makes it possible to tune IL–water interactions and thereby control the adhesion of liquid and solid aqueous precipitation to slippery coatings. This latter approach for controlling ice adhesion was tested in the present work.

The shear ice adhesion strength of SLIPS infused with the seven ILs listed in Table 1 was measured over 30 consecutive ice formation and detachment cycles. The position of the ice specimen on each coating was fixed so that changes in adhesion strength could be monitored within the same area and coating degradation caused by both the centrifugal loading and the IL removal with the detached ice could be evaluated.

**Figure 4:**
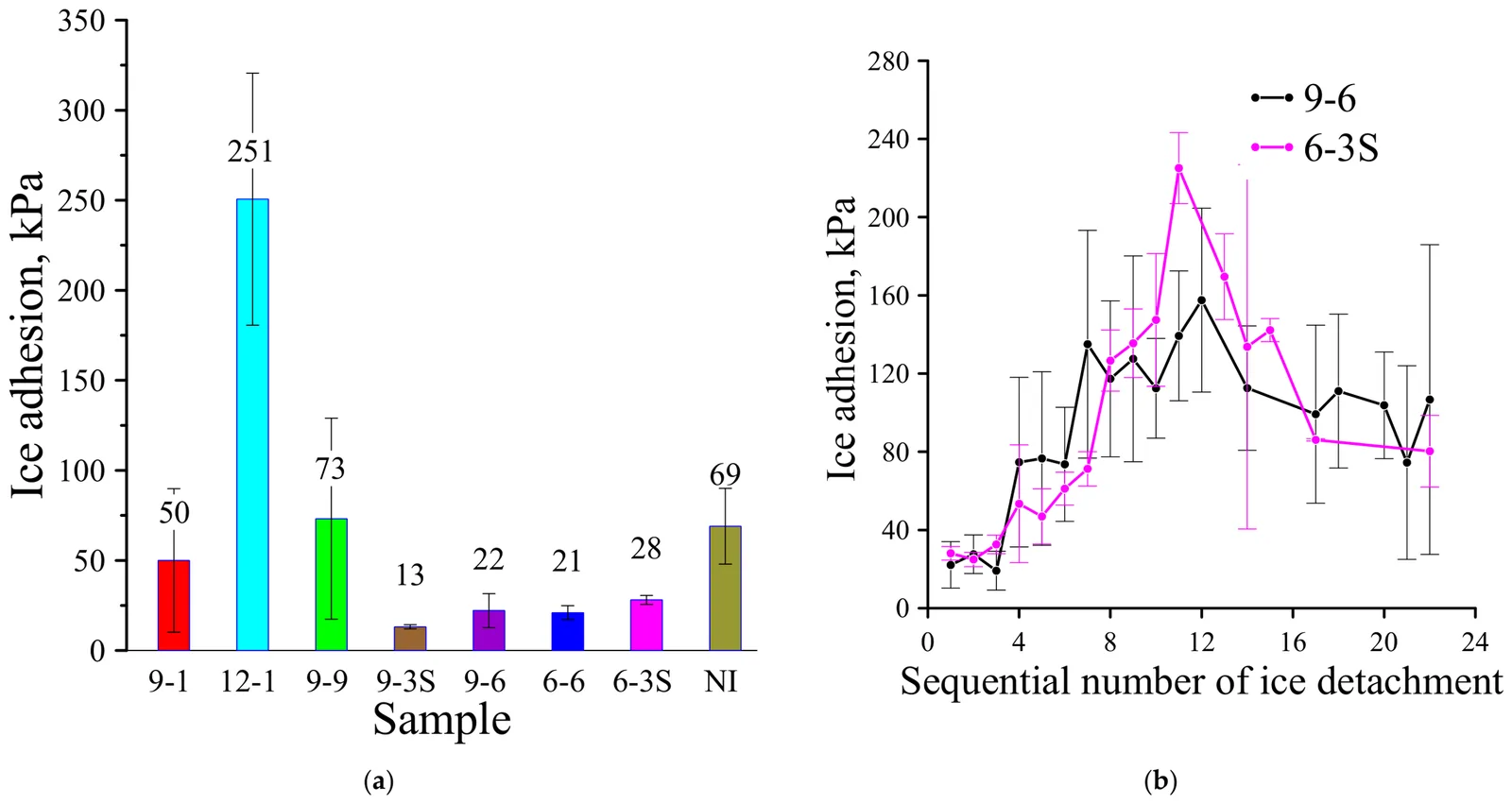
(**a**) Ice adhesion strength to the SLIPS samples with different ILs during the first icing–detachment cycle. NI denotes non-infused superhydrophobic coating. (**b**) Evolution of the shear ice adhesion strength of SLIPS infused with ILs 9-6 and 6-3S during consecutive ice formation and detachment cycles.

Ice adhesion was measured simultaneously for all coating types in a single experiment under identical temperature, humidity, and ice formation conditions. Even under nominally identical conditions, however, the scatter in adhesion strength among specimens of the same type can be substantial [46]. In each cycle, four replicate coatings of each type were tested. Figure 4a shows the ice adhesion strength to the samples during the first icing–detachment cycle. Low values were found for ILs that are poorly wetted by water. Thus, SLIPS with IL 9-3S exhibited the lowest ice adhesion strength during the first detachment cycle, while the water static contact angle on this coating was the highest. The behavior of SLIPS with ILs 9-9 and 12-1 again demonstrated the highest ice adhesion strength, which we attribute to complete freezing of the lubricating film for IL 12-1 or partial freezing for IL 9-9.

For context, shear ice-adhesion strengths below approximately 100 kPa are frequently described as icephobic in the literature, whereas values in the 10–20 kPa range represent particularly low adhesion and have been discussed in connection with passive ice removal [47]. These values should be viewed only as approximate benchmarks because measured ice adhesion depends strongly on test geometry, loading mode, temperature, ice preparation, and interfacial aging [48].

Representative changes in ice adhesion strength with cycle number for SLIPS infused with ILs 9-6 and 6-3S are shown in Figure 4b. Despite the scatter among the four replicate samples of each type, both ILs show the same general trend. The adhesion strength initially increases, reaches a maximum, and then decreases. Similar behavior was observed for SLIPS infused with ILs 9-1, 9-3S, and 6-6 (shown in Figure S1 in Supplementary Information).

For ILs 12-1 and 9-9, which were solid at *T* = −10 °C, the coating evolution was markedly different. Ice detached from these coatings through both adhesive and cohesive failure. Cohesive failure accounted for 81% of all detachments from the coating infused with IL 12-1 and 45% for the coating infused with IL 9-9.

**Figure 5:**
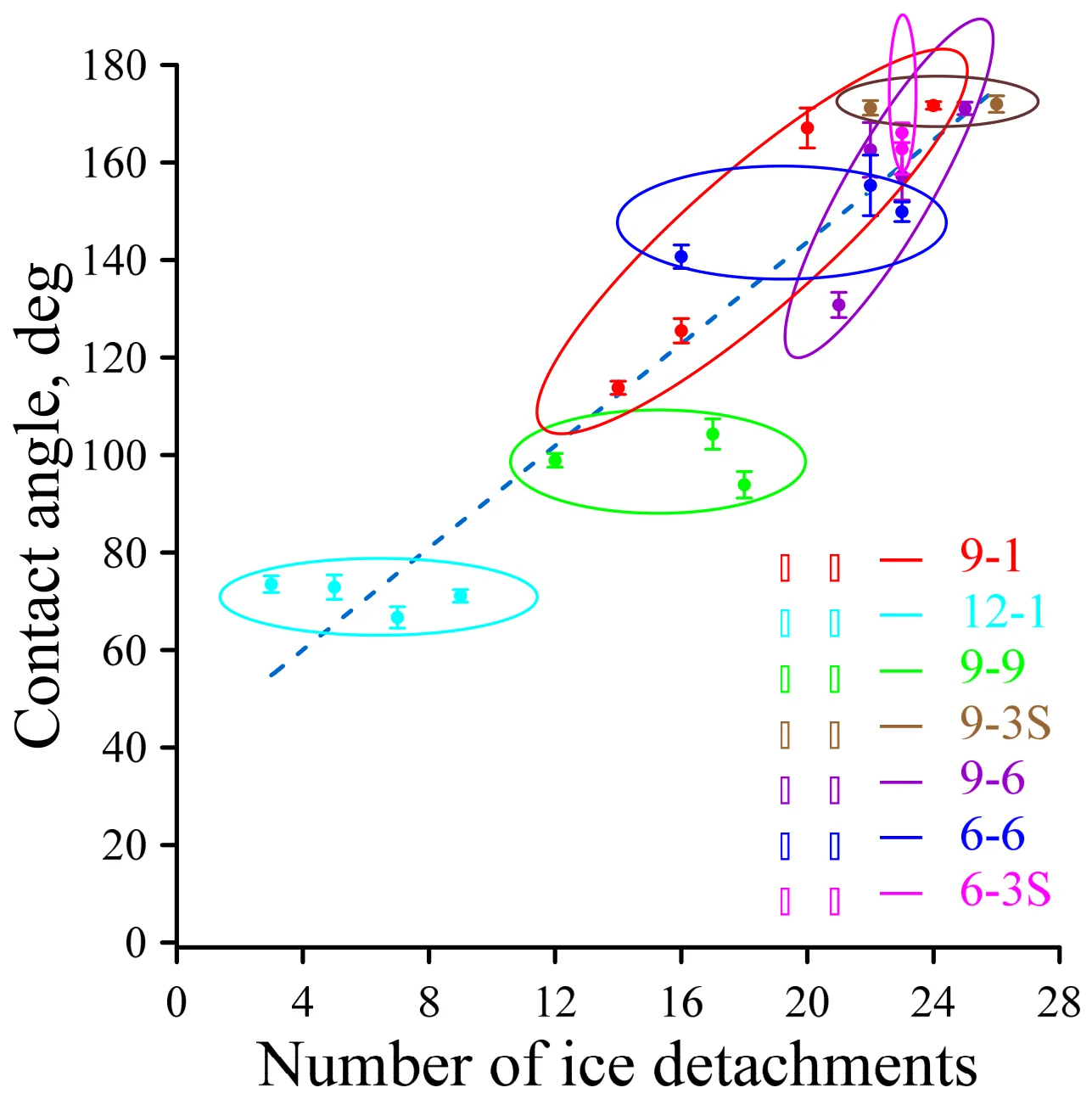
Water static contact angle in the central region of the samples exposed to ice as a function of the number of adhesive ice detachments. To facilitate comparison, the data for each IL are enclosed by an oval of the corresponding color. The dashed line is a linear fit to all experimental points and illustrates the correlation between the contact angle and hydrophilic lubricant removal with ice detachments.

To evaluate IL removal, water contact angles were measured after ice adhesion experiments directly within the ice-contact region and plotted versus an actual number of adhesive ice detachments. The data obtained for the different coatings are shown in Figure 5. To facilitate comparison, the data for each IL are enclosed by an oval of the corresponding color.

For ILs 9-1, 9-3S, 9-6, 6-6, and 6-3S, the contact angle generally increases with the number of detachments, consistent with removal of the hydrophilic lubricant from the superhydrophobic texture. After many cycles, the contact angles exceed 160° and reach values above 170° for ILs 9-1, 9-3S, and 9-6.

As noted in our recent studies, the ice adhesion strength on a rough coating, whether superhydrophobic or SLIPS, depends on the aging time of the ice/coating contact after ice formation [30,46]. This dependence is associated with the formation of ice bridges and their growth into the rough surface during recalescent ice crystallization. The bridges substantially increase the real ice/coating contact area and therefore ice adhesion. Ice-bridge formation is stochastic, and the fraction of grooves filled with ice varies among experiments depending on the duration of the recalescence stage. This behavior explains the large scatter in Figure 4.

When ice is detached after a prolonged period following its formation, the long-time evolution of ice bridges depends on the wettability of the texture walls into which the ice has grown. On a superhydrophobic surface, ice inside the grooves is metastable. Increasing the aging time causes sublimation of the ice bridges and reduces the contact adhesion strength [30,49]. Recent studies showed that after 15–20 h of interfacial aging, metastable bridges are removed from grooves with hydrophobic walls, and the adhesion strength is governed primarily by the equilibrium contact angle rather than the details of the texture [46]. For superhydrophobic coatings with water contact angles above 170°, the adhesion strength at *T* = −10 °C is below 30 kPa.

**Figure 6:**
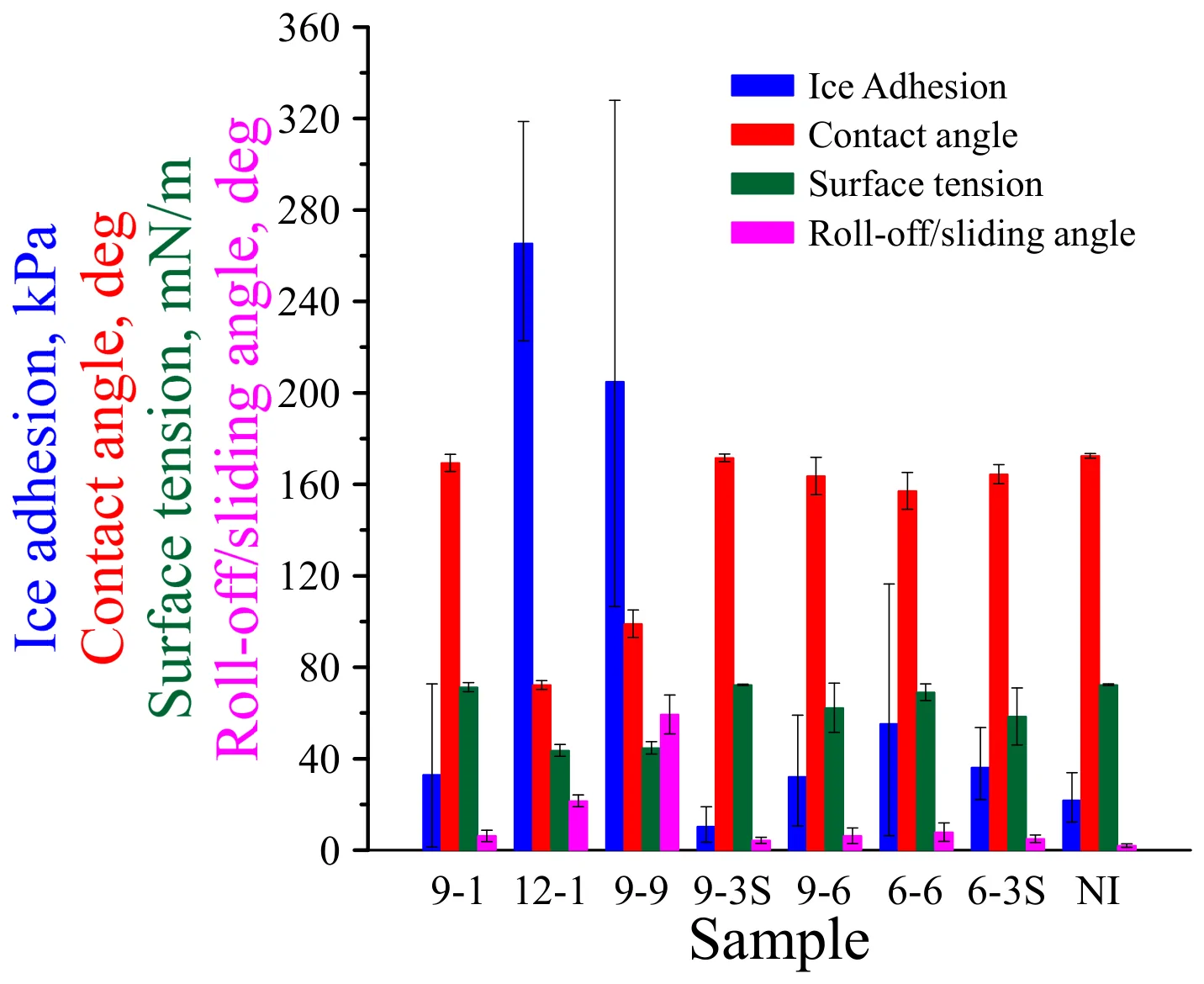
Relationship between shear ice adhesion strength after 20 h of aging, coating static contact angle, surface tension, and roll-off/sliding angle of the test water droplet. NI denotes non-infused superhydrophobic coating.

On hydrophilic walls, ice bridges are more stable and can persist in the pores for long periods. Therefore, both the mean adhesion to hydrophilic regions and the scatter in cyclic ice formation–detachment measurements remain high. The dependence of adhesion on aging time is weaker and is probably associated mainly with relaxation of interfacial stresses generated during crystallization. In Figure 6, the shear ice adhesion strength after 20 h of aging is juxtaposed with the water contact angle of the coatings and the surface tension of the test droplets. Note that the presented data correspond to the 30th ice formation–detachment cycle.

The data show that the minimum shear ice adhesion strength of 10.4 ± 8.6 kPa was obtained for the coating infused with IL 9-3S. After 30 ice detachments, this coating had a contact angle of 171.6 ± 1.6°, a roll-off angle of 4.4 ± 1.3°, and a droplet surface tension corresponding to water free of surface active impurities. Thus, after repeated cycling, the lowest adhesion was observed not for a surface that retained the original SLIPS state, but for a superhydrophobic substrate from which the infused IL had been almost completely removed by centrifugal loading and ice detachment.

A simultaneous decrease in the surface tension of the test droplet and in the contact angle after cycling indicated that some IL remained in the texture grooves. On the one hand, residual IL could be transferred to the test droplet during the wetting measurement. On the other hand, because it was hydrophilic (see Table 2), the residual IL created hydrophilic patches inside the grooves. These regions stabilized ice bridges, increased the effective ice–coating contact area, and increased the shear adhesion strength.

The highest adhesion was observed for coatings infused with IL 12-1 (265 ± 53 kPa) and IL 9-9 (205 ± 123 kPa). At *T* = −10 °C, these salts were solid and were therefore less efficiently removed from the coating both during ice detachment and under centrifugal force. The contact angles of these coatings remained close to those of the freshly prepared surfaces.

### 4.3. Mechanism of the Nonmonotonic Change in Adhesion

The observed nonmonotonic dependence of adhesion strength on cycle number during consecutive ice detachments after 1 h of ice–coating interfacial aging (Figure 4) is consistent with peculiarities of lubricant retention/removal during centrifugal loading and wettability variation. While the liquid IL layer remains sufficiently thick and the wetting films over the texture asperities are stable, ice slides over the IL under centrifugal loading, and only low shear stresses are required for detachment. Rupture of the IL wetting films over the asperity tops, however, shifts the system from hydrodynamic lubrication to boundary lubrication. Patches of direct contact then form between ice and the textured substrate, causing a sharp increase in shear strength. For a superhydrophobic coating, after ice bridges have sublimed, the total adhesion strength is low because of heterogeneous wetting and the small fraction of ice contacts with asperity tops. The situation differs for coatings that remain partly filled with IL. Their total ice–coating contact area is substantially larger because of the lower surface tension, resulting in effectively higher adhesion. Residual IL in the grooves stabilizes ice bridges, while the remaining IL layer is too thin to permit free ice sliding over the rough surface.

**Figure 7:**
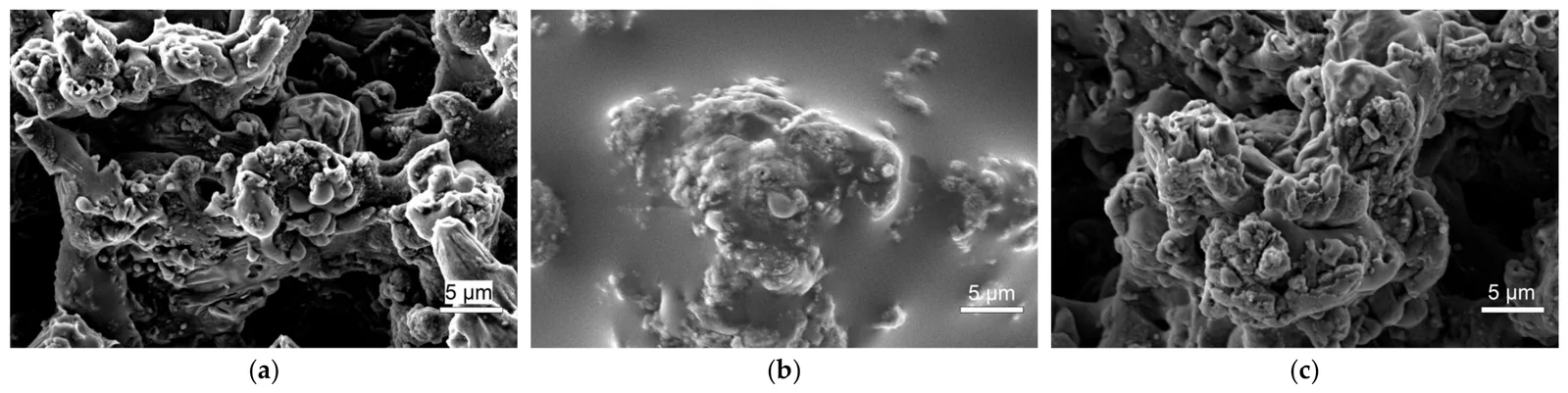
SEM images of coatings infused with ILs 9-1 (**a**), 12-1 (**b**), and 9-3S (**c**) after 30 ice detachment cycles.

Further removal of IL from the texture exposes a larger fraction of the superhydrophobic substrate. This decreases the ice–coating contact area, reduces the probability of stabilizing ice columns within the texture, and consequently lowers the adhesion strength. To test the proposed effect of residual IL on the ice–coating adhesion strength after 20 h of interfacial aging, the coating morphologies after 30 ice detachment cycles were examined by SEM (Figure 7). The selected images represent typical scenarios of the evolution of the lubricant content in the texture during repeated ice detachment: 9-1 represents strong depletion of a low-viscosity IL, 12-1 represents the strongly retained solidifying IL, and 9-3S represents the coating showing the lowest final adhesion and recovery of superhydrophobicity.

Indeed, the SEM images of coatings infused with ILs 9-1 and 9-3S show no appreciable amount of IL at the surface (compare with the freshly prepared samples in Figure 2). For these coatings, the surface tension of the test droplets is close to that of pure water, while the very high contact angles and low roll-off angles correspond to a superhydrophobic state.

In contrast, substantial filling of the texture remains for the coating infused with IL 12-1. For this coating, the surface tension of the test water droplet indicates the formation of a cloaking film over the droplet surface [20]. The contact angles remain close to those of freshly prepared SLIPS, and the ice shear adhesion strength remains high. These observations agree with the proposed mechanism: partial retention of a hydrophilic IL in the texture grooves stabilizes ice bridges and increases adhesion, whereas almost complete IL removal restores a superhydrophobic surface with lower adhesion after prolonged interfacial aging.

Direct three-dimensional quantification of ice bridge formation was out of the scope of the present study; the bridge-stabilization mechanism should therefore be regarded as a physically consistent interpretation supported by the observed wetting evolution and by previous interfacial-aging studies [28,46,49].

To briefly summarize this section, the nonmonotonic dependence can be interpreted as a transition through three interfacial states controlled by progressive depletion of the liquid IL layer. In the first state, the lubricant forms a sufficiently continuous and mobile layer over the texture, and ice detachment occurs predominantly by interfacial sliding. In the intermediate state, the lubricant film over the asperity tops becomes discontinuous losing effective liquid lubrication, whereas IL remains trapped in the grooves, retaining hydrophilic IL-rich regions, resulting in increased adhesion through ice bridging. Continued depletion finally exposes most of the underlying superhydrophobic texture, suppressing stable ice penetration into the grooves and causing the adhesion to decrease again.

## 5. Conclusions

Slippery textured coatings infused with ionic liquids (ILs) of different structures were investigated, and relationships were established among IL structure, thermophysical properties, lubricant-layer stability, and ice adhesion. Freshly prepared SLIPS were hydrophilic, with water static contact angles of 65.2–83.3°, while their sliding angles remained low, at 1.4–7.5°. Thus, water wetting did not prevent droplet removal at small coating inclinations.

The decrease in the surface tension of the test water droplets to 43.3–51.0 mN/m indicates IL transfer to the water–air interface and the formation of a cloaking film. This process accelerates lubricant depletion during repeated contact with aqueous precipitation.

Tests under repeated centrifugal loading showed that IL-layer stability is governed not only by substrate wettability, but also by lubricant viscosity and phase state at subzero temperature.

The main findings are as follows:

1. Low-viscosity ILs provide high droplet mobility but are removed from the surface more readily during centrifugal loading and cyclic ice detachment.
2. Lubricant retention in the texture does not necessarily reduce adhesion. ILs 12-1 and 9-9, which solidified at −10 °C, stabilized ice bridges in the pores and produced the highest ice adhesion even after prolonged interfacial aging.
3. The change in adhesion during cycling is nonmonotonic. Partial IL removal disrupts hydrodynamic lubrication and increases adhesion, whereas further exposure of the underlying superhydrophobic substrate again decreases the ice contact area and adhesion.
4. The lowest ice adhesion after 30 cycles was obtained for the coating infused with IL 9-3S. The surface had nearly recovered the superhydrophobic state characteristic of the substrate before its impregnation with IL, with a contact angle of 171.6 ± 1.6° and a roll-off angle of 4.4 ± 1.3°.

It should be emphasized that the substrate morphology was kept constant in the present study. Changes in roughness, pore dimensions, or texture geometry may modify lubricant retention, film stability, and ice penetration into the surface relief. Therefore, the absolute adhesion values and lubricant-depletion rates reported here should not be assumed to be morphology-independent.

The present ice-adhesion results are experimentally established at −10 °C and should not be extrapolated to other operating temperatures without accounting for the lubricant phase state and thermal history. For conventional durable SLIPS, a suitable IL should remain sufficiently mobile at the target temperature to preserve interfacial lubrication while having adequate viscosity, wetting affinity, and capillary retention to resist depletion. Crystallization within the texture is undesirable when the resulting immobile hydrophilic phase stabilizes ice penetration and bridging. Conversely, if lubricant depletion cannot be avoided, a low-adhesion underlying texture may provide a useful fail-safe state, as observed here for 9-3S. Thus, lubricant phase state, mobility, retention, and the wetting state of the exposed substrate should be considered jointly rather than optimized independently.

Note, however, that before large-scale practical use of candidate ILs, toxicological and biodegradation assessment is required.

## Figures and Tables

**Table 1:** Structures of the ionic liquids (ILs) investigated. The thermophysical properties of ILs 6-6 and 6-3S were characterized in our recent study [19] and are included here for the comparison.

IL Label	Abbreviated Notation	Cation Formula	*T*_g_ and *T*_m_ *	Cation Structural Formula
9-1	[C_9_C_1_im][NTf_2_]	C_13_H_25_N_2_^+^	*T*_g_ = −83 °C	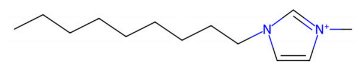
12-1	[C_12_C_1_im][NTf_2_]	C_16_H_31_N_2_^+^	*T*_g_ = −55 °C *T*_m_ = 16–24 °C	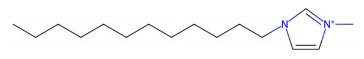
9-9	[C_9_C_9_im][NTf_2_]	C_21_H_41_N_2_^+^	*T*_g_ = −82 °C *T*_m_ = 8–20 °C	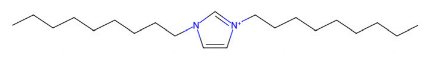
9-3S	[C_9_C_3_Si_2_Oim][NTf_2_]	C_20_H_43_N_2_OSi_2_^+^	*T*_g_ = −76 °C	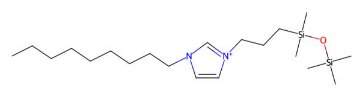
9-6	[C_9_C_6_im][NTf_2_]	C_18_H_35_N_2_^+^	*T*_g_ = −81 °C	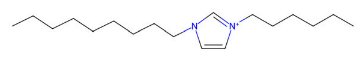
6-6	[C_6_C_6_im][NTf_2_]	C_15_H_29_N_2_^+^	*T*_g_ = −83 °C	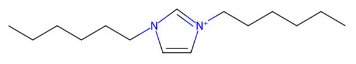
6-3S	[C_6_C_3_Si_2_Oim][NTf_2_]	C_17_H_37_N_2_OSi_2_^+^	*T*_g_ = −75 °C	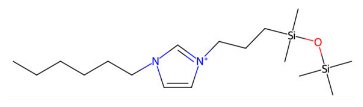

* Data from ref. [24].

**Table 2:** Surface tensions and static contact angles of used ILs on smooth hydrophobized mica and the water-wettability parameters of SLIPSs with corresponding ILs.

IL	Surface Tension, mN/m (*T* = 25 ± 0.3 °C)	IL Contact Angle on Hydrophobized Mica, Deg (*T* = 23 ± 2 °C)	Water Contact Angle on SLIPS, Deg (*T* = 23 ± 2 °C)	Water Droplet Sliding Angle, Deg	Water Droplet Surface Tension on SLIPS, mN/m
9-1	30.55 ± 0.05	76.8 ± 0.9	68.9 ± 0.4	4.0 ± 0.4	44.8 ± 1.2
12-1	30.68 ± 0.03	81.8 ± 0.7	65.2 ± 1.3	3.0 ± 0.5	43.3 ± 1.0
9-9	29.10 ± 0.04	75.1 ± 1.2	82.2 ± 0.5	7.5 ± 0.8	49.0 ± 0.2
9-3S	25.18 ± 0.03	77.0 ± 0.7	83.3 ± 0.5	4.5 ± 0.8	44.9 ± 0.8
9-6	29.08 ± 0.03	77.4 ± 0.7	79.9 ± 0.4	3.5 ± 0.6	47.2 ± 0.6
6-6	28.96 ± 0.01	77.2 ± 0.8	77.5 ± 1.1	1.4 ± 0.5	51.0 ± 1.2
6-3S	25.33 ± 0.03	70.5 ± 1.2	83.2 ± 1.2	2.4 ± 0.3	44.5 ± 0.7

## Data Availability

The original contributions presented in this study are included in the article and Supplementary Material. Further inquiries can be directed to the corresponding author.

## Supplementary Materials

The following supporting information can be downloaded at https://www.mdpi.com/article/10.3390/polym18172116/s1, Figure S1: Evolution of the shear ice adhesion strength of SLIPS infused with ILs 9-1, 9-3S, and 6-6 during consecutive ice formation and detachment cycles; Estimation of aerodynamic shear and centrifugal loading stresses. References [50,51,52] are cited in the Supplementary Materials.
