# Supplementary material for: When Lubricant Retention Increases Ice Adhesion: Ionic Liquids in SLIPS
Source: Polymers (Basel). 2026 Aug 31;18(17):2116. doi: 10.3390/polym18172116 (PMC13568457; doi:10.3390/polym18172116)
Supplement: Supplementary file 1 [file polymers-18-02116-s001.zip › polymers-4518980-supplementary.pdf]

Supplementary Information for the Article

When Lubricant Retention Increases Ice Adhesion: Ionic Liquids in SLIPS

Alexandre M. Emelyanenko [1], Timofei V. Golubitchenko [1], Vladimir G. Krasovsky [2], Kirill A. Emelyanenko [1] and Ludmila B. Boinovich [1,*]

[1] Frumkin Institute of Physical Chemistry and Electrochemistry, Russian Academy of Sciences, 119071 Moscow, Russia
[2] N.D. Zelinsky Institute of Organic Chemistry, Russian Academy of Sciences, 119991 Moscow, Russia
* Correspondence: boinovich@mail.ru:

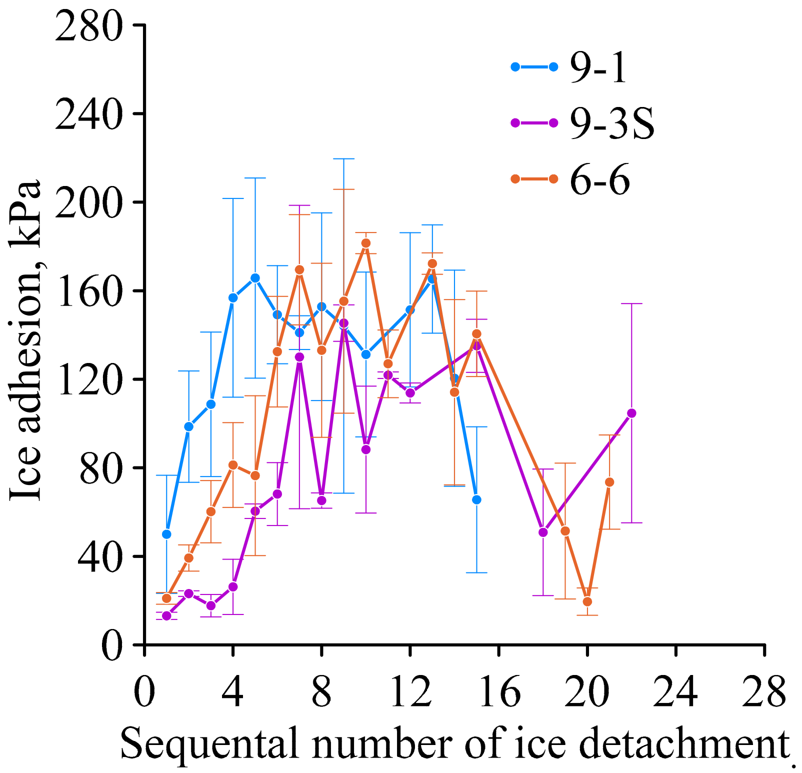

Figure S1. Evolution of the shear ice adhesion strength of SLIPS infused with ILs 9-1, 9-3S, and 6-6 during consecutive ice formation and detachment cycles.

Estimation of aerodynamic shear and centrifugal loading stresses

For aerodynamic flow, the wall shear stress can be expressed through the skin-friction coefficient as

$$\tau_w = C_f \, \rho_{air} \, V^2 / 2,$$

where C_f is the skin-friction coefficient, ρ_{air} is the air density, and V is the characteristic local air velocity. This definition is standard and is also used in the NASA CFPOST

 https://doi.org/10.3390/polym18172116

formulation for aerodynamic wall shear stress [1]. For a turbulent boundary layer, a commonly used flat-plate estimate is

$C_f = 0.074\ Re^{(-1/5)}$,

(where Re is Reynolds number), which is given, for example, in NASA aerodynamic performance calculations.

For modern wind turbines, characteristic blade-tip velocities are approximately 70–90 m/s [2]. Taking $\rho_{air} = 1.225$ kg/m^3, a characteristic length of 1 m, and the above turbulent flat-plate correlation gives Re number approximately 4.7×10^6 to 6.1×10^6, C_f approximately 0.0033, and consequently wall shear stresses of approximately 10–16 Pa.

For high-speed aircraft propellers, considerably higher local velocities can occur. NASA reports a design propeller tip speed of 243.8 m/s for advanced high-speed propellers [3]. Using a representative local chord length of 0.2–0.5 m gives Re approximately 3.3×10^6 to 8.3×10^6 and C_f approximately 0.0031–0.0037. The corresponding wall shear stress is approximately 110–135 Pa. Thus, the statement that aerodynamic skin-friction stresses relevant to wind-turbine blades and aircraft propellers are typically of the order of tens to about one hundred Pascals is consistent with standard boundary-layer estimates.

To quantify the centrifugal loading applied in our experiments, let us consider the following estimates. At 5000 rpm and a radial distance r = 0.12 m, the angular velocity is

$\omega = 2\ \pi \cdot n / 60 = 523.6$ rad/s,

and the centrifugal acceleration is

$a_c = \omega^2\ r = 3.29\times10^4$ m/s^2,

which corresponds to approximately 3350 g.

For a lubricant layer of thickness h on a surface oriented so that the centrifugal acceleration acts parallel to the surface, the centrifugal body force produces an interfacial shear stress that can be estimated as

$\tau_c = \rho_{IL} h\ a_c$,

where ρ_{IL} is the lubricant density. For a representative lubricant density of 1250 kg/m^3, this gives

- h = 1µm: τ_c approximately 41 Pa
- h = 2 µm: τ_c approximately 83 Pa
- h = 5 µm: τ_c approximately 206 Pa
- h = 10 µm: τ_c approximately 411 Pa.

Thus, even for a lubricant layer only approximately 1–5 µm thick, the centrifugal shear stress in our experiments is already comparable to, or greater than, the aerodynamic wall shear stresses estimated for wind-turbine blades and aircraft propellers. For thicker lubricant layers, the centrifugal loading becomes correspondingly more severe because τ_c increases linearly with film thickness.

This comparison also clarifies why the centrifugal experiment is particularly relevant to the present study. Aerodynamic loading acts primarily through the gas-liquid interface, whereas centrifugal loading acts as a body force throughout the lubricant volume. Nevertheless, both mechanisms drive lubricant redistribution and depletion along the textured surface. The present rotational test therefore should not be interpreted as an exact aerodynamic simulation, but rather as an accelerated mechanical stability test in which lubricant layers of micrometer-scale thickness are subjected to shear-driving stresses comparable to or exceeding those expected from aerodynamic skin friction in the intended application environments.

https://doi.org/10.3390/polym18172116

References

1. W.J. Fredericks, Conceptual design of a vertical takeoff and landing unmanned aerial vehicle with 24-HR endurance, in: AUVSI Unmanned Systems North America 2010, Denver, CO, USA, 2010, NASA Report NF1676L-10758.
2. J.I. Bech, C.B. Hasager, C. Bak, Extending the life of wind turbine blade leading edges by reducing the tip speed during extreme precipitation events, Wind Energ. Sci. 3 (2018) 729-748. https://doi.org/10.5194/wes-3-729-2018
3. L.C. Billman, C.J. Gruska, R.M. Ladden, D.K. Leishman, J.E. Turnberg, Large scale prop-fan structural design study. Volume 2: Preliminary design of SR-7, NASA Contractor Report 174993, Hamilton Standard, Windsor Locks, CT, 1988.

Disclaimer/Publisher's Note: The statements, opinions and data contained in all publications are solely those of the individual author(s) and contributor(s) and not of MDPI and/or the editor(s). MDPI and/or the editor(s) disclaim responsibility for any injury to people or property resulting from any ideas, methods, instructions or products referred to in the content.
